# Supplementary material for: ProteinShader: illustrative rendering of macromolecules
Source: BMC Struct Biol. 2009 Mar 30;9:19. doi: 10.1186/1472-6807-9-19 (PMC2672931; doi:10.1186/1472-6807-9-19)
Supplement: Additional file 1 — ProteinShader program without source code. This compressed file contains the complete ProteinShader program including associated libraries, but no source code. A README.txt file gives an overview of the ProteinShader distribution, and the index.html file in the help subdirectory has directions on getting started with the program as well as a set of tutorials. [file 1472-6807-9-19-S1.zip › ProteinShader-beta-0_9_4-binary/help/api/org/proteinshader/graphics/textures/package-summary.html]

org.proteinshader.graphics.textures (ProteinShader API)


|  |  |  |
| --- | --- | --- |
| **Overview** \| **Package** \| Class \| **Use** \| **Tree** \| **Deprecated** \| **Index** \| **Help** | |  |
| **PREV PACKAGE**   **NEXT PACKAGE** | **FRAMES**    **NO FRAMES**     **All Classes** |  |


---

## Package org.proteinshader.graphics.textures

Holds the classes needed for creating and managing OpenGL texture objects.

**See:**
  
          **Description**

| **Interface Summary** | |
| --- | --- |
| **ConfigReader** | Reads a configuration file to obtain a list of menu names and matching filenames for textures. |

| **Class Summary** | |
| --- | --- |
| **EqualSignConfigReader** | Reads a configuration file to obtain a list of menu names and matching filenames for textures. |
| **NamePair** | Stores a menu name and a file name for a texture. |
| **Texture** | Stores information on an OpenGL texture object. |
| **TextureFactory** | Reads texture files and creates OpenGL texture objects that can be used for real-time halftoning. |
| **TextureManager** | Uses a TextureFactory object to read textures from files and then stores Java Texture objects that have references to OpenGL texture objects and menu names that can be used for selecting the textures. |

## Package org.proteinshader.graphics.textures Description

Holds the classes needed for creating and managing OpenGL texture objects.
